# Abnormal Pavement Condition Detection with Vehicle Posture Data Considering Speed Variations

**DOI:** 10.3390/s24144555

**Published:** 2024-07-14

**Authors:** Qihua Zhan, Yuxin Ding, Tian Lei, Xiaohong Yin, Leyu Wei, Yunpeng Liu, Qin Luo

**Affiliations:** 1 College of Urban Transportation and Logistics, Shenzhen Technology University, Shenzhen 518118, China; 2210414008@email.szu.edu.cn (Q.Z.); 2310414019@stumail.sztu.edu.cn (Y.D.); yinxiaohong@sztu.edu.cn (X.Y.); luoqin@sztu.edu.cn (Q.L.); 2 Zhejiang HIKAILINK Technology Co., Ltd., Hangzhou 311100, China; weileyu@cethik.com (L.W.); liuyunpeng@cethik.com (Y.L.)

**Keywords:** pavement condition detection, inertial navigation system, vehicle speed, vehicle posture, machine learning

## Abstract

Pavement condition monitoring is an important task in road asset management and efficient abnormal pavement condition detection is critical for timely conservation management decisions. The present work introduces a mobile pavement condition monitoring approach utilizing low-cost sensor technology and machine-learning-based methodologies. Specifically, an on-board unit (OBU) embedded with an inertial measurement unit (IMU) and global positioning system (GPS) is applied to collect vehicle posture data in real time. Through a comprehensive analysis of both time domain and frequency domain data features for both normal and abnormal pavement conditions, feature engineering is conducted to identify how the most important features affect abnormal pavement condition recognition. Six machine learning models are then developed to identify different types of pavement conditions. The performance of different algorithms and the significance of different features are then analyzed. Moreover, the influence of vehicle speed on pavement condition assessment is further examined and classification models for different speed intervals are developed. The results indicate that the random forest (RF) model that considers vehicle speed achieves the best performance in pavement condition monitoring. The outcomes of the present work would contribute to cost-effective pavement condition monitoring and provide an important reference for pavement maintenance sectors.

## 1. Introduction

Poor pavement conditions, such as potholes, cracks, and unevenness resulting from untimely maintenance, have a significant negative impact on driving comfort, traffic efficiency, and safety. How to detect poor pavement conditions effectively and promptly has long been a key task in pavement management systems. The actual task of pavement condition detection can be time consuming and expensive, with traditional monitoring methods relying on manual site surveys and special patrol vehicles [1,2]. The Ministry of Transport of the People’s Republic of China reported that investments in pavement condition data collection and maintenance management in China will exceed CNY 258.7 billion during 2020–2025 [3]. Many transportation agencies may not collect pavement condition data on an annual basis for large portions of road networks due to the onerous work and high cost, which may further lead to improper pavement decisions at project and network levels as well as less-optimal allocation of agency funding and resources. Therefore, more emphasis has been placed on automated and cost-effective monitoring techniques in recent years to improve the efficiency of pavement condition data collection.

To improve the efficiency of pavement condition monitoring, researchers have proposed a variety of automatic pavement condition detection methods, which can be mainly divided into vision-based methods [4,5,6], LiDAR-based three-dimensional road structure construction methods [7,8], and acceleration sensor-based vibration response methods [9,10,11,12]. Among them, these methods based on vision and LiDAR scanning require the use of vehicles equipped with various professional sensors, which are usually expensive in terms of hardware and complex in terms of data processing [13]. In contrast, the acceleration sensor-based approach using an inertial measurement unit (IMU) provides a lower-cost and easier solution for pavement condition detection. A significant amount of research using vehicle posture data obtained from acceleration sensors has been conducted. Lei et al. [14] developed a cost-effective pavement condition monitoring system that employed a microcontroller and an IMU. The system measures a vehicle’s acceleration and angular velocity during driving and calculates the pavement condition parameters based on the collected data. Mishra et al. [15] used a cell phone with an acceleration sensor as the primary data acquisition unit and applied machine learning techniques to identify different types of pavement surfaces. Wu et al. [16] also used vehicle vertical vibration pulses for identifying roadway distresses and explored the performance of using time, frequency, and wavelet domain features. Basavaraju et al. [17] found that combining three-axis acceleration characteristics is more effective than using single-axis acceleration characteristics in pavement condition monitoring. To sum up, these studies show that the use of acceleration sensors to capture vehicle vibration data, combined with effective data mining or machine learning techniques, could provide an efficient way of pavement condition monitoring. In addition, considering different feature sources and feature domains could help improve the detection accuracy. However, it should be noted that considering multiple feature sources and feature domains could also result in greater computational costs and lower computational efficiency. Therefore, how to develop an effective model with appropriate feature engineering to balance between the accuracy and the computing efficiency becomes an important consideration.

While monitoring the pavement condition with acceleration sensors, the basic solution is to build the correlation between the characteristics of vehicle vibrations extracted from acceleration data with different types of pavement conditions through machine learning methods. One challenge aligned with such a solution is that the applicability may be affected by the vehicle speed. Previous studies have stated that vehicle speed is an important factor that may influence the performance of pavement condition detection using posture data. For instance, Wang et al. [18] conducted simulation-based experiments to explore the influence of different variables on roughness measurements using a smartphone. The results indicated that vehicle speed is one of the dominant factors that influence road roughness measurement. Another study [19] also estimated the dependence of the road roughness index on vehicle speed using vertical acceleration collected by mobile devices and demonstrated that, under the same conditions, the higher the vehicle speed, the higher the estimated road roughness index. Although those preliminary works reported that the influence of vehicle speed can be significant in pavement condition detection with posture data, the specific influence of vehicle speed on the performance of different machine-learning-based vehicle condition detection models is less discussed and still needs further exploration with field experiments.

To fill up the existing gaps, the present work therefore aims to deeply explore the potential of abnormal pavement condition detection with vehicle posture data and further specify the influence of vehicle speed on its performance. Specifically, an on-board unit (OBU), which is the vehicle-side communication terminal in the vehicle-infrastructure cooperative system (VICS), is applied as the data collection device for real-time vehicle posture data. Various machine learning and integrated learning algorithms are adopted to develop the most appropriate models for abnormal pavement condition detection. Moreover, sensitivity analysis is conducted to further examine the influence of vehicle speed on pavement condition monitoring. Major contributions of the present work are summarized as follows:

(1) The present work developed six machine learning models for pavement condition detection and fully examined the influence of vehicle speed on model effect through incorporating speed as a feature as well as training the model separately based on speed intervals. Subsequently, pavement condition detection models for different speed intervals were built, which greatly enhances the applicability and reliability of the machine-learning-based pavement condition detection solution with posture data.
(2) The present work also discussed the importance of considering different types of vehicle posture data and further combined a suitable feature screening method to balance between model performance in terms of accuracy and efficiency in feature engineering. Specifically, a sensitivity analysis of different feature groups was conducted and the shapely additive explanations (SHAP) method [20] was further applied to extract the most significant features in model development.
(3) The present work further examined the possibility of using OBU as a mobile sensing tool for dynamically identifying the pavement condition. The information on the distribution of pavement anomalies obtained by OBU could provide data support for early warning of anomalous pavements and roadway quality assessment services, which effectively expands the application scenarios of VICS technology and contributes to better promotion of the VICS system in dynamic infrastructure monitoring as well as traffic safety improvement.

The structure of this paper is summarized as follows. In Section 2, we introduce the evolution of pavement condition detection based on sensor technology. Section 3 introduces the data scenario of this study and the data processing procedure of the pavement condition classification model. Section 4 specifically examines the performance of different algorithm types and the accuracy of the model under different speed conditions. Section 5 summarizes the applicable scenario of this research.

## 2. Literature Review

The increase in road mileage and vehicle numbers has raised the demand for more precise and frequent assessment of road conditions by road management departments. In response to the requirement of efficiency and cost in road condition monitoring, various automatic pavement condition detection methods have been developed.

With the advancement of sensing technology and data processing technology, specialized road detection vehicles installed with specific sensors were first applied. For instance, responsive road roughness meters were mounted on the vehicle to measure the longitudinal profile of the pavement. The basic idea is to obtain the vertical distance per kilometer traveled through a dual integral of vertical acceleration value [21]. Chen et al. [22] used a digital video recorder (DVR) mounted in front of the vehicle to conduct pavement anomaly detection using support vector machine (SVM) classifiers and bag of words models for feature extraction. Some scholars also applied a laser scanner on vehicles for road damage identification [23] or multiple sensors for pavement monitoring [24]. However, the need for these advanced sensors and their high price limits the promotion of such systems for large-scale applications. Moreover, the performance of such systems based on vision and LiDAR scanning techniques may be affected by weather conditions. Therefore, more cost-effective mobile monitoring techniques are still needed.

Considering the above needs, a number of more cost-effective ways of monitoring pavement conditions have been proposed. These methods utilize devices such as IMUs and mobile phones equipped with accelerometers, GPS, and gyroscopes as sensing devices for pavement condition monitoring. The basic idea is to process and analyze the collected vehicle vibration data using threshold-based or machine learning methods to map the corresponding pavement condition. Threshold-based methods mainly empirically design feature variables and corresponding thresholds to recognize different pavement conditions and manually adjust the thresholds according to the actual application effect of the method [13]. For example, Mednis et al. [25] designed several combinations of thresholds to recognize potholes in pavements, and the results showed that the combination obtained by using 0.2 gravitational acceleration as the threshold and offset of vertical acceleration as the feature variable had the highest recognition accuracy. However, in the real driving process, it is usually difficult to determine reliable threshold or judgment variables considering the variation in the vehicle type and driving speed. As a comparison, machine learning methods can automatically establish the relationship between feature variables and target labels, thus providing a more effective way of identifying pavement conditions. For instance, Anaissi et al. [26] proposed a network-level road condition detection system that utilizes two statistical features of vertical acceleration to detect pavement anomalies through SVM algorithms. Egaji et al. [27] extracted 42 features from the vehicle vibration data and applied several machine learning models to identify potholes. The results showed that the random forest (RF) model with hyperparameters adjusted by grid search achieved the highest recognition accuracy. Jang et al. [28] also used a neural network to detect the pavement condition and fused the trajectories of multiple vehicles to determine the abnormal location. These studies demonstrated that machine learning methods could provide a reliable solution for pavement condition detection. However, the performance of different machine learning methods in abnormal pavement detection based on vehicle vibration data still needs further exploration.

To improve the accuracy and efficiency of pavement anomaly recognition with machine learning methods, researchers also conducted studies from the perspective of feature engineering. For instance, Anaissi et al. [26] considered both vehicle vertical acceleration and lateral acceleration data to extract features for the identification of pavement anomalies and potholes. Celaya et al. [29] extracted a total of 42 time domain features from vehicle three-axis acceleration and three-axis angular velocity data obtained by on-board accelerometers and gyroscopes for pavement speed bump detection. Some scholars [30,31] extracted both time domain and frequency domain features from accelerometer and gyroscope data and applied SVM to identify different types of pavement conditions. These studies proved that considering features in different dimensions based on vehicle posture data could achieve a positive effect in improving the performance of pavement condition monitoring using machine learning methods. One concern aligned with the expansion of the feature set is the increase in computing complexity caused by higher feature dimensions. Thus, several attempts have been made to apply feature selection algorithms to obtain the optimal combination of features [32]. As can be seen, how to construct the most comprehensive feature set while at the same time combining a suitable feature screening method to balance between model performance in terms of accuracy and efficiency is a significant task.

Another concern aligned with machine-learning-based pavement condition monitoring using vehicle posture data is the influence of vehicle driving speed. Specifically, previous studies have pointed out that vehicle speed may influence the effectiveness of pavement condition recognition. For instance, Li et al. [33] experimentally found that a high driving speed (within 30 km/h to 60 km/h) would increase the vibration of the *Z*-axis acceleration compared to a lower speed (less than 30 km/h). Loprencipe et al. [34] further reported that the index used for roughness evaluation should be characterized by different speed ranges and proved that different recognition results may be achieved for the same pavement quality under different speed ranges. These studies mainly calculated the pavement roughness index through a theoretical model based on acceleration data and further evaluated the influence of vehicle speed on the evaluation results. In a machine-learning-based pavement condition detection scenario, the potential influence of vehicle speed on the performance of different detection models still lacks comprehensive discussion.

Therefore, the major purpose of the present work is to fully discuss the performance of abnormal pavement condition detection based on various machine learning methods using vehicle posture data considering vehicle speed variations. Specifically, field driving experiments under different speed ranges on different pavement conditions were conducted to explore the influence of speed variations on pavement condition detection performance through machine learning methods. Moreover, comprehensive feature engineering was conducted to explore the significance of different data features. The results of the present work could provide valuable insights for improving the accuracy and efficiency of pavement condition monitoring systems to ensure safety.

## 3. Materials and Methods

**Figure 1:**
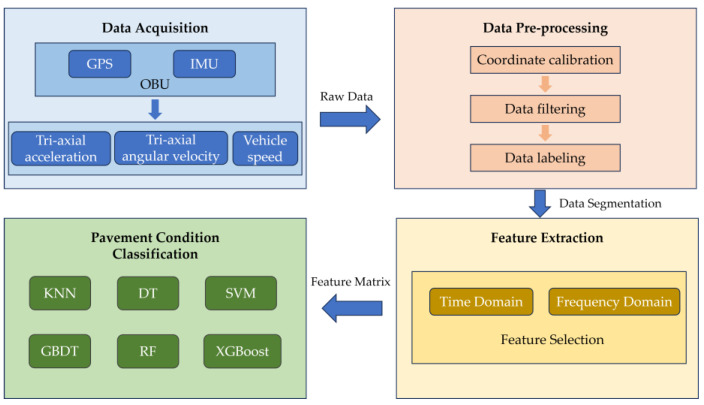
Flowchart of the proposed method.

This paper proposes a pavement condition detection method based on vehicle posture data, as shown in Figure 1. Firstly, field driving test was conducted for data acquisition using OBU mounted on the test vehicle. After the test, a comprehensive data preprocessing procedure was conducted to prepare a reliable data set for model training and development. Then, the feature extraction module was applied to construct the comprehensive features inputs for classification models. Finally, six machine learning and integrated learning methods, including SVM, decision trees (DT), k-nearest neighbor (KNN), RF, gradient boosting decision tree (GBDT), and extreme gradient boosting (XGBoost) were applied to recognize abnormal pavement condition. The process of data acquisition, data preprocessing, feature extraction, and machine learning approaches used in this study are introduced in detail as follows.

### 3.1. Data Acquisition

In this study, an OBU (embedded with an IMU sensor and GPS module) was used for data collection. The device adopted in the present work was produced by Zhejiang HIKAILINK Technology Co. as the vehicle terminal part of VICS. With the embedded IMU sensor, tri-axis acceleration and tri-axis rotation rate data can be obtained. Meanwhile, with the high-precision GPS, vehicle trajectory data can be monitored. These raw data were transmitted at a 10 HZ frequency.

**Figure 2:**
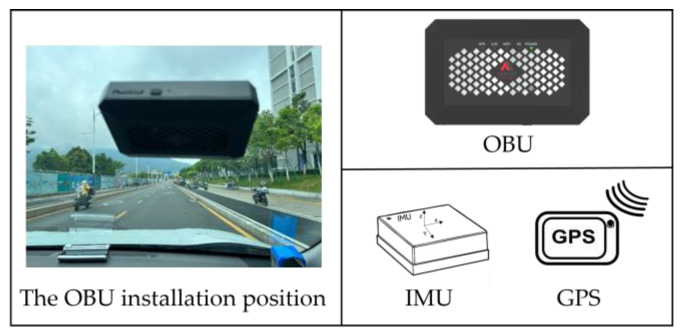
The description and installation of OBU.

During the process of data acquisition, the OBU was placed on the upper front window of the vehicle, as shown in Figure 2. This position ensures stable transmission of the GPS signal and 4G signal of the OBU and prevents continuous data packet loss. While collecting data with the OBU, we also used the mobile phone camera to record the pavement conditions during the driving process. According to the video recording data, the pavement condition is mainly divided into two categories: normal pavement conditions and abnormal pavement conditions. Specifically, a normal pavement condition is defined as a pavement segment that looks flat and smooth and no obvious depressions or bumps are observed, while an abnormal pavement condition represents pavement segments where there are obvious cracks, depressions, or bumps that contain most of the road disease problems.

**Figure 3:**
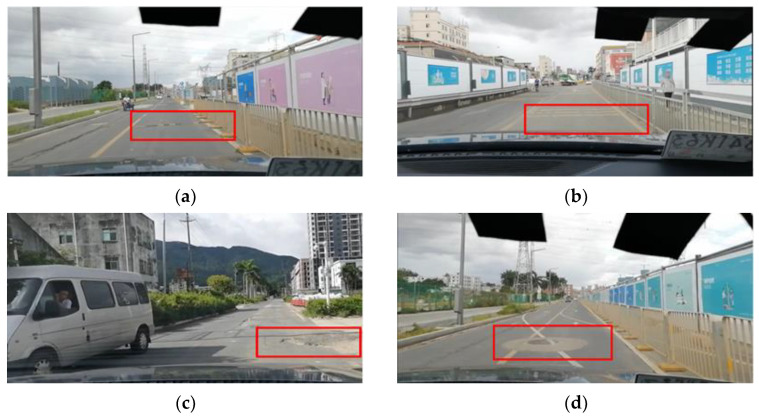
Abnormal pavement conditions in the test: (**a**) speed hump; (**b**) deceleration line; (**c**) pothole; (**d**) manhole cover.

Consider that factors that affect vehicle posture data during driving may include pavement conditions, road slope, external environmental conditions (such as wind and weather effects), speed variations, and driving behavior [35]. In order to minimize the possible effects of other factors, the data acquisition process was controlled in this study to ensure that the collected vehicle posture data were mainly affected by road surface conditions and speed variations. Firstly, the test was conducted on a windless, sunny day to avoid interference from the environment. Secondly, while driving the BYD SUV test vehicle, the driver maintained a steady driving style to prevent data noise caused by aggressive driving behavior. Finally, the test was carried out on urban roads with gentle slopes, covering various typical types of abnormal pavement samples, such as speed bumps, deceleration lines, potholes, and manhole covers, as shown in Figure 3. The data collection test was carried out in Pingshan District, Shenzhen.

### 3.2. Data Preprocessing

**Figure 4:**
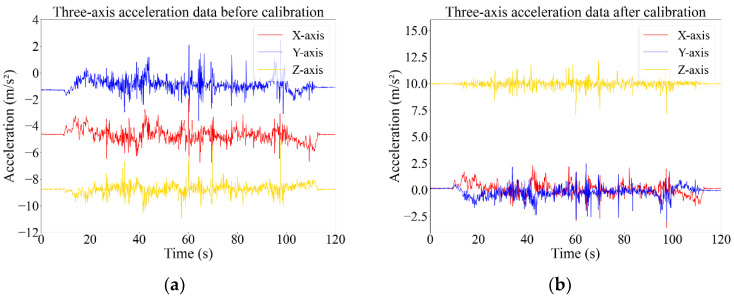
Tri-axis acceleration before and after coordinate transformation: (**a**) acceleration before calibration; (**b**) acceleration after calibration.

Before analyzing the characteristics of the vehicle posture data, it is essential to preprocess the data to ensure the stability and reliability of the collected information. The main work includes coordination transformation and data filtering. In this research, we have adopted Euler angle conversion, as outlined in related studies [36,37], to transform the raw data from the sensor’s coordinate system to the vehicle’s coordination. As illustrated in Figure 4, this transformation aligns the acceleration data of the X and Y axes, while the *Z*-axis acceleration value should be close to the gravity acceleration coefficient, thereby validating the efficacy of the data conversion.

**Figure 5:**
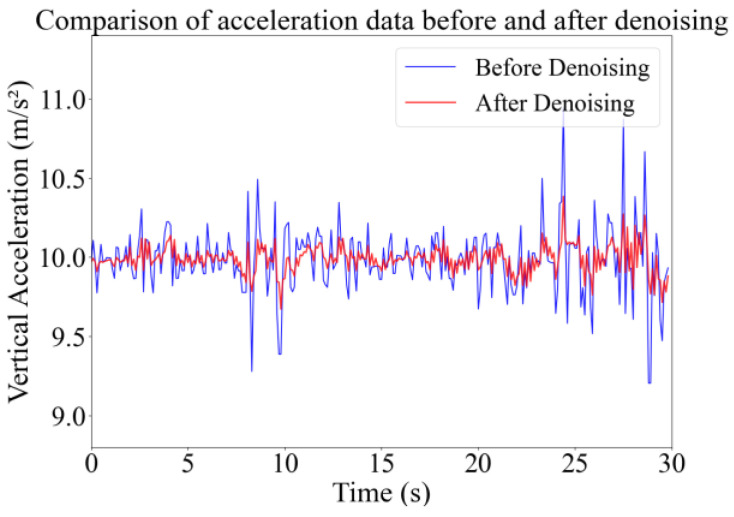
Vertical acceleration before and after noise filtering.

During the data collection process, due to measurement device errors, environmental interference, and data transmission inaccuracies, noise reduction is required to approximate the data closer to reality. In the present work, a first-order low-pass filter was used for data noise elimination. The low-pass filter has a smoother response to signal changes, which allows more signal frequencies to pass through the filter without attenuation [37]. Through setting the appropriate filter coefficient, it is suitable for making the data signal smooth while at the same time capturing the trend of posture data changes. The performance of the low-pass filter for noise filtering is depicted in Figure 5.

To recognize abnormal pavement conditions at a specific location, the data series after preprocessing are first divided into small pieces, which are taken as the time window for pavement condition evaluation. In the present work, we adopted a one-second time window, which generally corresponds to a ten-meter length road section for pavement condition evaluation. After the data collection experiments, a total of 6511 segments were extracted from the collected dataset, including 4419 segments for normal pavement and 2092 segments for abnormal pavement.

The potential bias in model predictions toward the majority class due to sample class imbalance was considered, which adversely affects the model’s ability to identify anomalous conditions [38]. Given that the ratio of normal to anomalous type samples collected is approximately 2:1, to ensure the accuracy of model training and the adequacy of sample size, this study duplicated the entire set of anomalous samples to achieve the balance between these two different types. Ultimately, the study amassed a total of 8602 samples for the purpose of model training and evaluation, which includes 4184 samples of anomalous pavement condition and 4418 samples of normal pavement condition.

### 3.3. Feature Extraction

Feature engineering is a key step for machine learning model development. The previous literature mainly focused on a limited number of features considered to be highly discriminative and has not fully explored all possibilities. To achieve the best classification results, the present work also examines the influence of different feature sources and combinations on the model training outcomes [39,40]. As mentioned before, the obtained data includes the vehicle’s tri-axis acceleration, tri-axis angular velocity, position, and vehicle speed. Most studies have solely utilized vertical acceleration to build their models. Some studies [29,30,31] also demonstrated that the simultaneous utilization of tri-axis acceleration and tri-axis angular velocity can lead to better training results, as this method considers more sufficient discriminative features. Therefore, this study utilized both tri-axis acceleration and tri-axis angular velocity to extract features for classification models. To further explore the potential influence of speed on pavement condition recognition, the present work also considered vehicle speed to be an important feature for assessing pavement condition.

**Figure 6:**
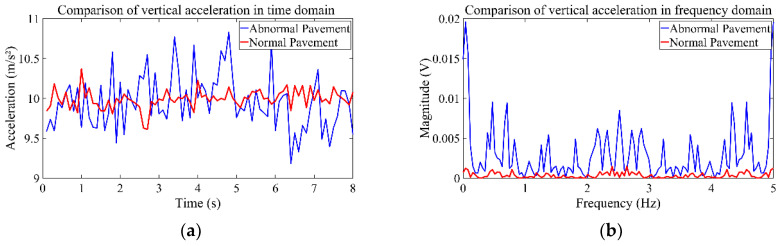
Vertical acceleration in time and frequency domain: (**a**) vertical acceleration in time domain; (**b**) vertical acceleration in frequency domain.

A comparison of vertical acceleration of normal and abnormal pavement conditions in the time domain and frequency domain is shown in Figure 6. As can be seen from Figure 6, the distribution characteristics of vertical acceleration pulse vary for the time domain and frequency domain. To perform the conversion from the time domain to the frequency domain, we employed the Fast Fourier Transform algorithm that comes bundled with Python. We considered that higher feature dimensions may increase the data processing complexity, which may increase the processing time or influence the prediction efficiency [36,41]. It was therefore necessary to screen the features first to reduce the influence of irrelevant or redundant features on model efficiency. We then adopted the SHAP method [20] for feature screening in order to compare the effects of different feature sources on the model recognition effect. Taking the *Z*-axis acceleration features as an example, the SHAP method was applied to screen 36 features extracted from the *Z*-axis acceleration data, and the top 20 highly significant feature variables based on the average absolute SHAP value of each feature were selected. Specifically, 12 features were chosen from the time domain, including standard deviation, mean, maximum, minimum, extreme deviation, corrected mean, root mean square, shape, margin, kurtosis, peak, and skewness. In addition, 8 features were selected from the frequency domain perspective, including signal amplitude area, spectral energy, frequency standard deviation, root mean square free frequency, maximum amplitude, bandwidth, mean frequency, and center of the spectrum. This allowed us to extract features from the seven-dimensional sample data and obtain a total of 140 feature variables. It should be noted that the weight of each feature variable was determined by the corresponding algorithm.

### 3.4. Machine Learning Methods

After the data preprocessing and feature extraction were completed, a suitable classification model was selected to classify pavement conditions. Compared to deep learning models, traditional machine learning models have the advantages of low computational cost, ease of interpretation, strong universality, and better performance for small samples, which are more suitable for the data in this paper. This paper focuses on the classification performance of machine learning models for acceleration, angular velocity, and vehicle speed. Referring to relevant studies [38,39], it is proposed to use SVM, DT, KNN, RF, GBDT, and XGBoost to build the model.

The model trained based on different algorithms was selected and the confusion matrix was used to evaluate the training effect of the model. According to the confusion matrix, the accuracy of the model for the training set and the validation set as well as the accuracy of the model for different pavement types, recall, and F1 score can be obtained. Among them, accuracy is the evaluation of the model as a whole, precision reflects the correct ratio of the model to the positive class of the prediction results, recall reflects the ratio of correct identification of the model to the positive class of the prediction results, and the F1-score is the harmonic average of precision and recall.

All algorithms were implemented and tested on a Dell Playbox G15 laptop (Dell, Xiamen, China) running the Windows11 Home Edition operating system and equipped with an AMD® Core™ R7-5800H CPU @ 3.20 GHz (AMD, Santa Clara, CA, USA) and 16 GB RAM (Samsung, Seoul, Republic of Korea). Data preprocessing was achieved using functions and a programming environment on MATLAB R2023b, while the machine learning classification algorithm was implemented using a classifier provided by Scikit-Learn on a Python 3.7.6 environment.

## 4. Results and Discussion

In this section, the paper discusses and analyzes various classifiers and the dimensions of feature variables, with a special focus on the impact of vehicle speed on the recognition of pavement conditions. Initially, the study utilizes all features related to vehicle posture, as mentioned in the preceding text, to compare the performance of different classifiers. Given the limited research on the individual contributions of the six dimensions of vehicle posture data, namely the three-axis acceleration and three-axis angular velocity data, we have designed six datasets to analyze the contributions of features across these six dimensions. Lastly, considering the varying responses of vehicles to different pavement conditions under varying speed conditions, this study has conducted experiments to validate the impact of speed on the model’s recognition accuracy.

For each experiment, the datasets were randomly divided into training and testing sets in a 7:3 ratio. In the experiments that analyzed the influence of characteristic variables in different dimensions, the classifiers with the best performance were selected to establish the models, respectively. The accuracy of the test set and the training set as well as the precision, recall, and F1 score of different pavement types in the test set were used to evaluate the performance of the model [42].

### 4.1. Evaluation of Classifiers

In this research, a comparative analysis of the training efficacy of six supervised learning algorithms for pavement condition classification was conducted, including three basic classifiers and three ensemble classifiers based on decision tree algorithms. The results are shown in Table 1.

The results demonstrate that all six classification algorithms achieved an accuracy exceeding 80% in identifying various types of pavement conditions. Notably, ensemble learning algorithms outperformed basic classifiers, particularly with the RF and XGBoost algorithms, which surpassed 90% accuracy in the training set. Among these, the RF algorithm exhibited the most robust performance, attaining the highest accuracy of 92.04% in the training set. Additionally, in the test set, models trained using the RF algorithm achieved an accuracy of 88.01% in recognizing normal pavement conditions and 84.96% for abnormal pavement conditions.

This superior performance is likely due to the high feature count in this study, where the ensemble method of averaging multiple basic classifiers’ results outperformed the approach of training new weak classifiers based on the negative gradient information of the current model’s loss function. This finding is consistent with previous research that ensemble methods can effectively improve the performance of classifiers by combining multiple models. Furthermore, results showed that all six classifiers were more effective in classifying normal pavements compared to abnormal pavements. For instance, the accuracy of the RF algorithm in identifying normal pavements was 3.07% higher than that for abnormal pavements, indicating a more complex distribution of sample features in abnormal pavements, making them more challenging to accurately identify.

In conclusion, the pavement condition classification models developed using machine learning algorithms all achieved an accuracy rate exceeding 80%. The classifier trained with the RF algorithm demonstrated the best recognition performance, with an accuracy of 91.60%. A comparative analysis of the model’s performance revealed a greater proficiency in identifying normal pavements, likely due to the more complex conditions present in abnormal pavements, where mildly damaged anomalies are difficult to distinguish from the normal state.

### 4.2. Evaluation of Feature Sets

Based on the vehicle posture data collected by the OBU, including the three-axis acceleration and three-axis angular velocity information of the vehicle, we have established a model to test the effect of using all the features in the vehicle posture data of these six dimensions to characterize the pavement conditions. In this section, we aim to further explore the impact of features from different dimensions on the model’s recognition performance. The process entailed initially generating six distinct datasets by sequentially eliminating feature variables from various dimensions. Subsequently, models for pavement condition recognition were developed for each dataset. These models were then compared with the model trained using the complete feature set to assess the influence of different feature dimensions on the model’s recognition accuracy. All models were trained using the RF algorithm and the results are shown in Table 2.

The results indicated that excluding any one parameter category led to a decrease in the model’s recognition accuracy, thereby affirming the positive impact of each parameter category on accuracy. By comparing the extent of the decrease in accuracy, as shown in parentheses in Table 2, it was possible to determine the relative importance of different source features. Notably, the *Z*-axis acceleration was the most critical, with a decrease in training set accuracy of 0.5%. This was followed by the *Y*-axis acceleration, which saw a 0.22% drop in accuracy. The *X*-axis angular velocity and *X*-axis acceleration were next in importance, with respective decreases of 0.20 and 0.19% in training set accuracy. The *Y*-axis and *Z*-axis angular velocities were found to be similarly important, both resulting in a 0.12% reduction in accuracy.

In conclusion, all parameter data had a significant influence on the model’s pattern recognition abilities, with longitudinal acceleration and lateral acceleration having the greatest impact on the algorithm’s recognition performance. Furthermore, incorporating a broader range of source features in the sample was beneficial for the algorithm’s recognition capabilities, resulting in more accurate outcomes.

### 4.3. Vehicle Speed Sensitivity Analysis

To further examine the potential influence of vehicle speed on pavement condition detection, a sensitivity analysis on vehicle speed was also conducted. In this section, two verification methods were designed. One was to incorporate vehicle speed as an input feature in model development, while the second approach was to train RF models separately based on speed intervals. The major process and results of these two methods are discussed as follows. To examine the influence of vehicle speed, a set of comparative experiments was designed. The difference was to consider speed-related factors in features including standard deviation, mean, maximum, minimum, range, and root mean square of the vehicle speed as feature variables or not. The results of the models trained with and without speed-related features are shown in Table 3. The results indicated that incorporating speed features led to an improvement in the model’s accuracy. Specifically, the precision of the model in identifying abnormal pavement conditions increased from 84.94% to 85.30% when speed features were incorporated. Such results further validate that speed is an important factor affecting model performance in detecting pavement conditions. However, the efficiency of the model after considering the speed feature was affected at the same time and the training time of the model increased from 2.21 s to 2.42 s. Therefore, there is a need to explore more efficient ways regarding the pavement condition recognition model considering speed.

To further verify the influence on model performance, we also developed different models based on different speed intervals. Specifically, the overall dataset was divided into five subsets based on the mean speed of each sample. The composition of each dataset is detailed in Table 4. Models were then developed using each dataset. The final recognition results were compared with those from models that did not consider speed stratification and the findings are presented in Table 5. This comparative approach underscored the significance of speed as a variable in improving the precision of models designed to identify pavement anomalies.

It can be seen in Table 4 that the data samples for normal and abnormal pavement types in different groups are imbalanced. In order to avoid the phenomenon that the imbalance of categories in some speed intervals may cause the model to be biased toward the majority of the class samples, in the training phase of the RF algorithm model in this study, the category weight parameter was set to “balance” so that the category with a small number of samples was given a larger weight. The performance of different RF models developed based on different speed intervals are shown in Table 5. As can be seen in Table 3 and Table 5, the overall performance of the RF-based abnormal pavement detection model is better than the model developed with speed as features, especially at lower speed intervals (within 45 km/h). It is obvious that model accuracy can reach over 92% when the speed interval is 0–30 km/h. This may be attributed to the random feature selection process inherent in the RF algorithm during training, which may have resulted in some classifiers not considering speed-related features, thereby diminishing the impact of speed on the training effectiveness. In contrast, actively training models based on different speed levels directly emphasizes the importance of the speed variable, resulting in more accurate models due to the distinction in vehicle vibration levels across various speed intervals.

In addition, the experimental results again demonstrated that the speed magnitude has a significant effect on the effectiveness of the model. Specifically, the model is seen to be more effective in the 0–45 km/h speed interval but the model accuracy decreases when the speed exceeds 45 km/h. This suggests that this method of pavement condition monitoring using posture data is more stable in lower speed ranges. The reason for this may be due to the fact that vehicles are more prone to significant vibration under higher speed conditions [33], so the causes of vehicle vibration are more complex, leading to more difficulty in identifying segments of data due to anomalous pavement conditions. Another possible reason for the above problem is that the vibration data is collected at a frequency of 10 Hz and the vibration signal change may not be well captured when the vehicle speed is too fast. It should also be noted that, for testing groups with imbalanced data samples, the difference between the recognition precision for normal and abnormal types is relatively small. For instance, the recognition precision for normal and abnormal types for the testing group of over 60 km/h is 85.81% and 83.05%, respectively. Such results indicate that the imbalanced data samples for different categories do not cause too much bias for model recognition performance.

In summary, both experiments designed in this study confirm that vehicle speed is a key parameter for pavement condition classification models. Specifically, training the recognition model with different speed ranges was more effective. Moreover, it is indicated that the performance of the pavement condition detection model based on machine learning methods using vehicle posture data could be more stable in lower speed ranges than in high-speed ranges.

## 5. Conclusions

The present work deeply investigated the potential for abnormal pavement condition detection with vehicle posture data using machine learning methods considering the influence of vehicle speed. Specifically, vehicle posture data during driving on road sections with different pavement conditions were collected through OBU. Then, a comprehensive data preprocessing was conducted to obtain a reliable data set. After that, different feature sets and feature domains from posture data were extracted for model construction. Finally, six different machine learning algorithms were developed for abnormal pavement condition detection. A sensitivity analysis was also conducted regarding the influence of different feature sets and the influence of speed variations. The major conclusions obtained in the present work are summarized as follows.

Firstly, through comparing the performance of six different machine learning methods for recognizing abnormal pavement conditions, it is observed that the RF algorithm performs the best in classifying different pavement conditions. Overall, the RF, GBDT, and XGBoost algorithms achieve better accuracy than DT, SVM, and KNN. Following this, the present work further explored the importance of different feature sources in vehicle posture data using the RF algorithm. The results indicate that features extracted from the *Z*-axis (vertical) acceleration and *Y*-axis (lateral) acceleration data have the greatest influence on the recognition of pavement conditions. Moreover, the effect of considering vehicle speed on model performance is deeply examined through sensitivity analysis. Compared with the method of incorporating speed as input features, training the model separately based on speed intervals achieved better performance in recognizing abnormal pavement conditions. It is further verified that vehicle speed plays an important role in road traffic monitoring using vehicle posture data. Specifically, when the vehicle speed is within 45 km/h, the accuracy of the classifier is relatively higher than that at a higher speed. Specifically, the accuracy of the classifier is relatively high when the vehicle speed is up to 45 km/h and the accuracy of the model in distinguishing between abnormal and normal pavement conditions decreases when the vehicle speed exceeds 45 km/h. Similar to the findings in reference [34], more normal pavement conditions are recognized as abnormal pavement conditions under high-speed situations. Such results indicate that the solution of monitoring pavement conditions using vehicle posture data may work better at a lower speed than at a high speed.

The findings of this study could be applied as a warning about abnormal pavement conditions and to assess road quality to improve transportation safety, and in the meantime, it could provide an important reference for the development of cost-effective pavement condition monitoring techniques. It should also be noted that the current work roughly categorizes pavement conditions into two types (normal and abnormal), while more complex research into more detailed pavement conditions (such as speed bumps, road seams, and potholes) should be considered in future work. It is also necessary to cover more data collection scenarios to verify the robustness of the model under the influence of factors such as road gradient and driving behavior. Furthermore, future research could also consider the effect of vehicles traveling at higher speeds and discuss the potential impact of different vehicle types on the recognition effectiveness of the model.

## Figures and Tables

**Table 1:** Comparison of the performance of different classifiers.

Classifier	Accuracy of Training Set	Accuracy of Testing Set	Type	Precision	Recall	F1-Score
DT	88.67%	81.29%	Normal	81.70%	82.13%	81.92%
			Abnormal	80.84%	80.38%	80.61%
SVM	82.87%	81.90%	Normal	81.92%	85.74%	83.79%
			Abnormal	83.99%	79.82%	81.86%
KNN	86.40%	81.71%	Normal	82.53%	81.91%	82.22%
			Abnormal	80.86%	81.51%	81.18%
GBDT	85.63%	84.58%	Normal	85.65%	84.23%	84.94%
			Abnormal	83.48%	84.95%	84.21%
XGBoost	90.32%	85.28%	Normal	86.45%	84.76%	85.60%
			Abnormal	84.08%	85.83%	84.94%
RF	92.04%	86.48%	Normal	88.01%	85.44%	86.70%
			Abnormal	84.94%	87.59%	86.24%

**Table 2:** Comparison of the performance within different feature sets.

Feature Sets	Accuracy of Training Set	Accuracy of Testing Set	Type	Precision	Recall	F1-Score
All Feature	92.04%	86.48%	Normal	88.01%	85.44%	86.70%
	(0.00%)	(0.00%)	Abnormal	84.94%	87.59%	86.24%
Exclude X-Acc	91.85%	86.28%	Normal	87.73%	85.36%	86.53%
	(0.20%)	(0.19%)	Abnormal	84.82%	87.27%	86.03%
Exclude Y-Acc	91.75%	86.26%	Normal	87.62%	86.11%	86.86%
	(0.30%)	(0.22%)	Abnormal	85.46%	87.03%	86.24%
Exclude Z-Acc	91.58%	85.97%	Normal	87.42%	85.06%	86.23%
	(0.47%)	(0.50%)	Abnormal	84.51%	86.95%	85.71%
Exclude X-rotate	91.65%	86.28%	Normal	87.96%	85.59%	86.76%
	(0.40%)	(0.20%)	Abnormal	84.85%	87.51%	86.16%
Exclude Y-rotate	91.83%	86.36%	Normal	87.85%	85.74%	86.78%
	(0.22%)	(0.12%)	Abnormal	84.85%	87.35%	86.08%
Exclude Z-rotate	91.83%	86.36%	Normal	87.87%	85.36%	86.60%
	(0.22%)	(0.12%)	Abnormal	84.85%	87.43%	86.12%

**Table 3:** Comparison of results on velocity characteristics.

Feature Domain	Accuracy of Training Set	Accuracy of Testing Set	Training Time (s)	Type	Precision	Recall	F1-Score
Include Speed	92.53%	86.59%	2.21	Normal	87.86%	85.89%	86.86%
				Abnormal	85.30%	87.35%	86.31%
Exclude Speed	92.04%	86.48%	2.42	Normal	88.01%	85.44%	86.70%
				Abnormal	84.94%	87.59%	86.24%

**Table 4:** A description of the data on the speed of the test vehicle.

Speed Range (km/h)	Normal	Abnormal	Total
0~15	375	96	471
15~30	1467	1324	2791
30~45	1889	1710	3599
45~60	571	730	1301
>60	116	324	440

**Table 5:** Comparison of model performance for different speed intervals.

Speed Range (km/h)	Accuracy of Training Set	Accuracy of Testing Set	Type	Precision	Recall	F1-Score
0~15	96.05%	92.96%	Normal	94.64%	96.36%	83.87%
			Abnormal	86.67%	81.25%	83.87%
15~30	96.21%	92.42%	Normal	92.86%	76.47%	83.87%
			Abnormal	92.31%	97.96%	95.05%
30~45	94.40%	86.28%	Normal	88.28%	84.83%	86.52%
			Abnormal	84.29%	87.84%	86.03%
45~60	96.48%	83.33%	Normal	84.49%	82.97%	83.73%
			Abnormal	82.14%	83.72%	82.92%
>60	96.75%	84.14%	Normal	85.81%	76.88%	81.10%
			Abnormal	83.05%	89.91%	86.34%

## Data Availability

The original contributions presented in the study are included in the article; further inquiries can be directed to the corresponding author.
